# Case Report: Abdominal Wall Abscess as First Clinical Sign of Jejunal Perforation After Blunt Abdominal Trauma

**DOI:** 10.3389/jaws.2024.13682

**Published:** 2024-11-26

**Authors:** M. Martínez-López, M. Verdaguer-Tremolosa, V. Rodrigues-Gonçalves, M. P. Martínez-López, M. López-Cano

**Affiliations:** ^1^ General and Digestive Surgery Department, Hospital Universitari Vall d’Hebrón, Barcelona, Spain; ^2^ Department of Surgery, UD of Medicine of Vall d’Hebron, Universitat Autònoma de Barcelona, Abdominal Wall Surgery Unit, General and Digestive Surgery Department, Hospital Universitari Vall d’Hebrón, Barcelona, Spain

**Keywords:** abdominal trauma, retromuscular abscess, mesh, abdominal wall infection, biosynthetic

## Abstract

**Aim:**

To discuss extended retrorectal abscess secondary to blunt abdominal trauma as a cause of abdominal wall (AW) infection and impairment.

**Methods:**

According to the CARE checklist, we describe a rare case of blunt abdominal trauma with late diagnosis of jejunal perforation with an abscess that extensively dissected the retromuscular space.

**Results:**

A 65 years-old female patient experienced multiple traumas after a traffic collision. Ten days after admission, the patient presented with swelling in the right abdomen. CT scan showed localised pneumoperitoneum and extensive collection affecting the right retrorectal space, reaching the ribs and preperitoneal space. Urgent laparotomy was performed and jejunal perforation with biliary peritonitis and extraperitoneal extension with dissection of the right retrorectal space were found. Intestinal resection with anastomosis was then performed. Exhaustive lavage of the cavity and retromuscular space with debridement of the necrotic posterior rectus lamina was required. Retrorectal drainage was placed. Primary closure of the aponeurosis was achieved using a small-bites technique with a slowly absorbable monofilament suture. Due to the weakness of the abdominal wall, an absorbable biosynthetic mesh impregnated with gentamicin was placed onlay. Negative pressure therapy was applied to the closed wound. Patient received antibiotics and CTs showed favourable evolution. No infectious complications or incisional hernia were reported after 12 months of follow-up.

**Conclusion:**

No cases of blunt trauma causing extensive AW infection have been reported in the literature. Whilst rare, this should be considered in traumatic patients. Our experience shows that they can be managed with surgical drainage and absorbable meshes can be considered in cases of fascial loss.

## Introduction

Blunt abdominal trauma can cause a wide spectrum of injuries, ranging from superficial contusions to deep visceral damage. Severe abdominal wall lesions can pose a challenge for surgeons; they not only compromise patients’ survival and postoperative complications, but also their quality of life. These injuries are still misdiagnosed and inefficiently managed, and evidence for their treatment is scarce in the literature, with no previously reported cases of blunt trauma causing extensive AW infection. According to the CARE checklist, we describe a rare case of blunt abdominal trauma with late jejunal perforation causing an extraperitoneal abscess that extensively dissected the right retromuscular space.

## Case Description

A 65-year-old female, with no medical history, except for a previous hysterectomy, suffered multiple traumatic injuries during a severe high-intensity traffic collision. After resuscitation and complete standardized initial evaluation, computed tomography (CT) was used to exclude other abdominal injuries. Additionally, she presented multiple contusions, multiple costal fractures, sternum fracture and right superior extremity fracture (Injury Severity Score of 21).

**FIGURE 1:**
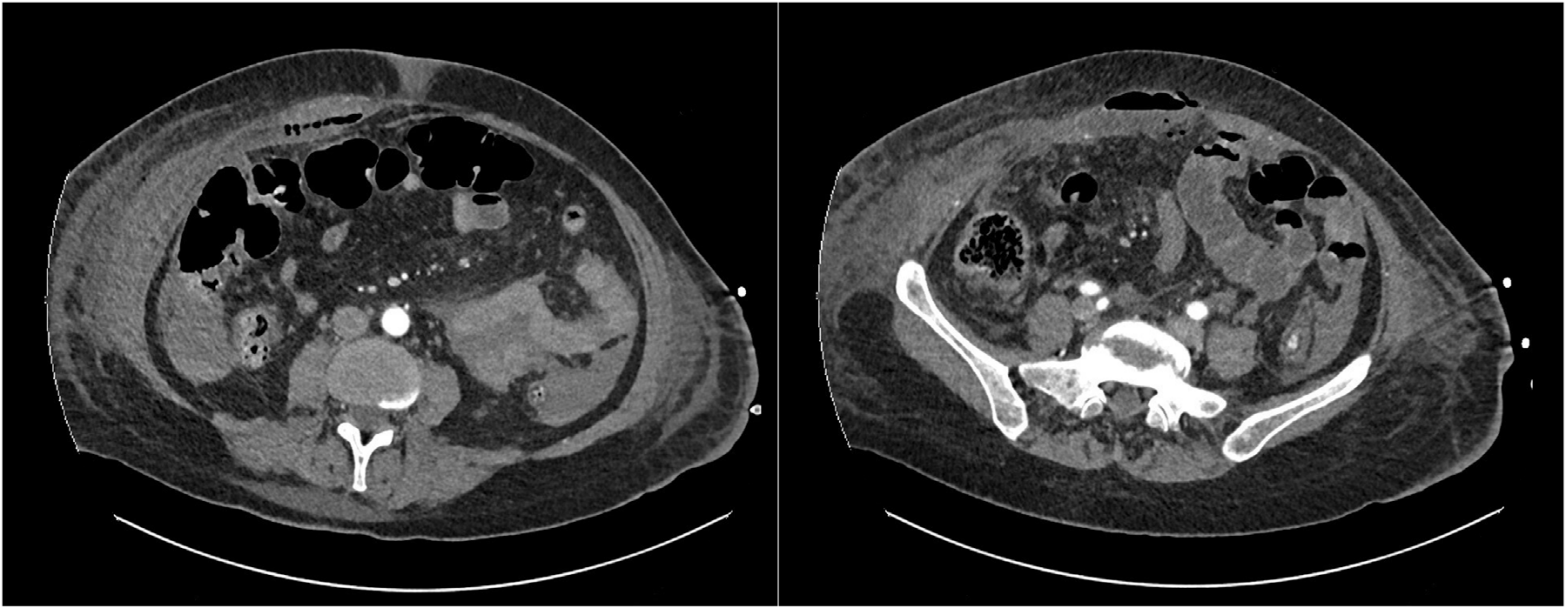
Abscess extension through the right retrorectal space with local pneumoperitoneum.

On the 10th day after admission to the critical care unit, she presented with fever and swelling in the right abdominal region. On physical examination, the patient presented with pain and abdominal guarding upon palpation of right abdominal region. Blood tests showed acute-phase reactants, and CT revealed the presence of local pneumoperitoneum with an extensive abscess in the right retromuscular (retrorectal) space, reaching up to the level of the ribs with preperitoneal involvement, all of which were related to a delayed jejunal perforation with fistulization to the retromuscular space (Figure 1).

Emergent laparotomy was performed. After positioning a wound protector, biliary peritonitis was found, with extensive involvement and dissection of the right retromuscular space due to delayed post-traumatic jejunal perforation.

**FIGURE 2:**
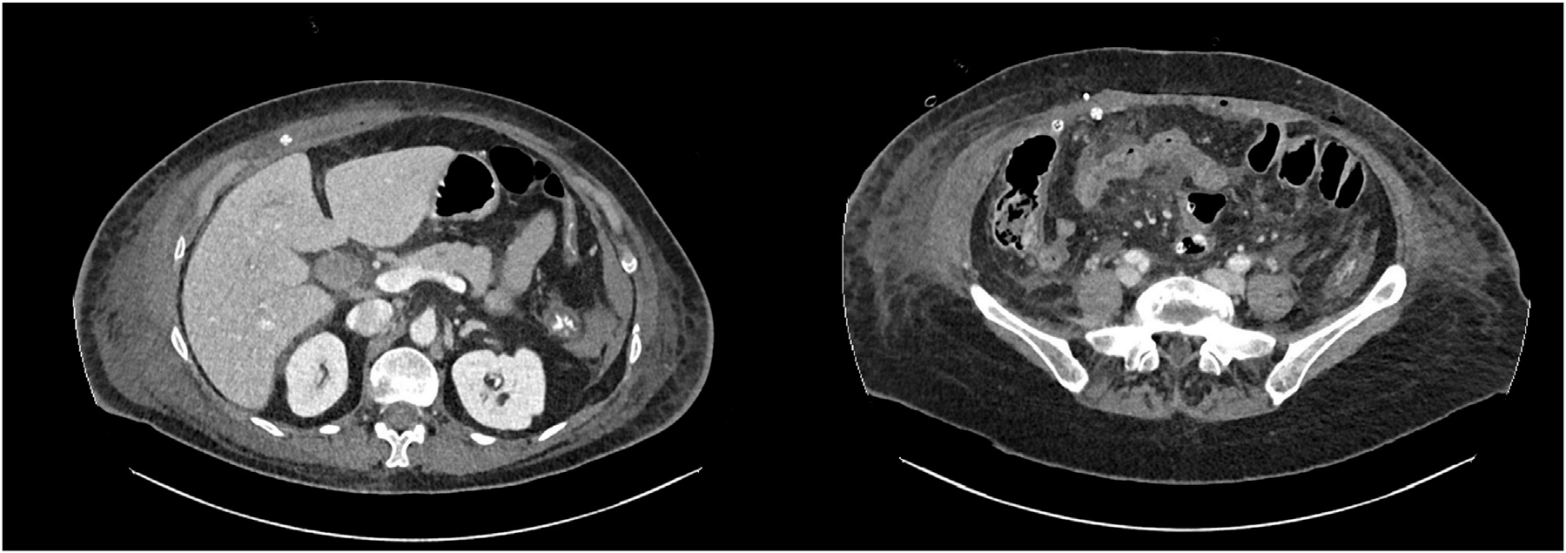
Postoperative CT scan with retromuscular and subcutaneous drainage above the mesh.

Intestinal resection with an ileo-ileal hand-sewn anastomosis was performed along with meticulous debridement of the necrotic tissue involving the abdominal wall, including part of the right posterior rectus sheath. Exhaustive lavage of the cavity and retromuscular space with saline and antiseptics was performed. A drainage tube was placed in the retromuscular space (Figure 2).

Primary closure of the aponeurosis was achieved using a small-bite technique [1] with a slowly absorbable monofilament. Due to the weakened state of the aponeurosis and the high risk of developing an incisional hernia and burst abdomen in this context, after an exhaustive lavage, an absorbable biosynthetic mesh (BioA^®^ 9 × 15 cm) impregnated with gentamicin was placed onlay to reinforce the midline. Subcutaneous drainage was placed. Negative pressure wound therapy was applied to reduce the risk of wound infection and dehiscence [2].

The patient received targeted antibiotic therapy based on the intraoperative culture results. Subsequent CT scans demonstrated favourable evolution. The patient was successfully discharged 52 days after surgery because of respiratory complications secondary to COVID-19. No signs of infection or incisional hernia were detected after 12 months of follow-up, and patient referred a completely recovered quality of life.

## Discussion

Abdominal wall impairment can occur in up to 9% of blunt abdominal trauma [3]. The energy of the impact transmitted to the abdominal wall can generate injuries, such as contusions, lacerations, and bone fractures at the costal or pelvic level. Rapid deceleration or compression forces can generate a shearing or stretching effect on the fascia and muscles of the abdominal wall, which could lead to a greater risk of traumatic hernias or disruption of retroperitoneal structures. These lesions can present with acute and exacerbated symptoms, such as pain, ecchymosis, or rebound tenderness, but late presentations include indiscernible symptoms complicating its diagnosis and increasing the patient’s morbimortality.

It has been reported that in most cases, the presence of a severe abdominal wall injury is accompanied by an intra-abdominal visceral injury and in those situations prompt surgical exploration is mandatory [4]. Even when visceral injury is excluded, it seems relevant to manage myofascial defects during the same hospitalization to prevent intestinal strangulation and occlusion. The question remains when the best time for intervention is and which type of procedure should be considered [5, 6].

A complex abscess of the retromuscular space is a very rare complication of blunt abdominal trauma with a challenging diagnosis owing to the latency of symptoms and their subtlety [7]. The high index of suspicion required in these cases, even in mild trauma, can determine the functionality of the anterior abdominal wall [5]. However, few studies have addressed abdominal wall injuries beyond hernia defects following contusion, and no data have specifically evaluated the possible infectious complications or its diagnosis and management. These traumatic injuries can be difficult to diagnose even with modern imaging techniques [8]. Nonetheless, in this case, an imaging tool, such as CT, was crucial for the diagnosis [9].

Regarding the treatment, despite the absence of robust evidence in the emergency context, a primary tension-free closure according to previously described techniques should be performed whenever possible [1, 10]. Mesh augmentation has also been described in these cases [11, 12]. Staged closure or mesh augmentation can be considered in cases of extensive tissue loss, where tension-free closure cannot be assured [13]. Contaminated or devitalized tissues and intestinal fistulas increase the difficulty of closing the abdominal wall and exacerbate the risk of incisional hernia [14]. Concerns regarding infection arise when mesh augmentation is suggested to prevent evisceration or incisional hernia, especially in contaminated or dirty surgeries. Even in hostile environments, some types of meshes have been proven to be safe and reduce the risk of incisional hernias [15, 16]. In a recent randomized clinical trial by Rosen et al., both synthetic meshes and biological implants demonstrated similar safety profiles in the context of a single-stage repair of contaminated ventral hernias [17]; however, synthetic meshes demonstrated a superior 2-year hernia recurrence risk compared with biologic mesh [18].

Despite the limitations of this case report, our data contribute to the scant existing literature, outlining the wide range of clinical presentations and therapeutic strategies of a complex infection after blunt abdominal trauma with the rare presentation of a dissecting retromuscular abscess due to visceral injury. Early detection and prompt surgery, along with the use of adequate prosthetic materials and abdominal wall surgical techniques, yield improved results in terms of morbimortality.

In conclusion, although blunt trauma causing extensive AW infection is rare, it should be considered in a traumatic context. This case highlights the importance of clinical suspicion, prompt intervention, and precise surgical technique with mesh use to improve patient outcomes and preserve abdominal wall integrity. Further investigation is necessary to establish management and prognostic factors for these patients.

## Data Availability

Publicly available datasets were analyzed in this study. This data can be found here: Data about the patient is confidential and available in the local informatic healthcare system.
